# Chemical delithiation and exfoliation of ${\mathrm{Li}}_{x}{\mathrm{CoO}}_{2}$

**DOI:** 10.1016/j.jssc.2014.08.011

**Published:** 2014-12

**Authors:** Angelika Basch, Liliana de Campo, Jörg H. Albering, John W. White

**Affiliations:** a Center of Sustainable Energy Systems, The Australian National University, Canberra ACT 0200, Australia; b Institute of Physics, University of Graz, A-8010 Graz, Austria; c Applied Mathematics, The Australian National University, Canberra ACT 0200, Australia; d Graz University of Technology, A-8010 Graz, Austria; e Research School of Chemistry, The Australian National University, Canberra ACT 0200, Australia

**Keywords:** Li-ion Battery, Neutron diffraction, Chemical delithiation, Intercalation, Rietveld refinement

## Abstract

Progressive chemical .delithiation of commercially available lithium cobalt oxide (${\mathrm{LiCoO}}_{2}$) showed consecutive changes in the crystal properties. Rietveld refinement of high resolution X-ray and neutron diffraction revealed an increased lattice parameter *c* and a reduced lattice parameter *a* for chemically delithiated samples. Using electron microscopy we have also followed the changes in the texture of the samples towards what we have found is a critical layer stoichiometry of about ${\mathrm{Li}}_{x}{\mathrm{CoO}}_{2}$ with *x*=1/3 that causes the grains to exfoliate. The pattern of etches by delithiation suggests that unrelieved strain fields may produce chemical activity.

## Highlights

• Chemical delithiation of ${\mathrm{Li}}_{x}{\mathrm{CoO}}_{2}$ showed consecutive changes in crystal properties.
• We performed X-ray as well as neutron diffraction on delithiated material.
• Crystal analysis revealed an increased *c* and a reduced *a* for delithiated samples.
• Electron microscopy revealed changes in the texture for ${\mathrm{Li}}_{x}{\mathrm{CoO}}_{2}$ (*x*=1/3).

## Graphical abstract

The effect of chemical dedoping of ${\mathrm{Li}}_{x}{\mathrm{CoO}}_{2}$ leads to a significant change in the (003) peak and to exfoliation for *x*=1/3.

**Figure:**
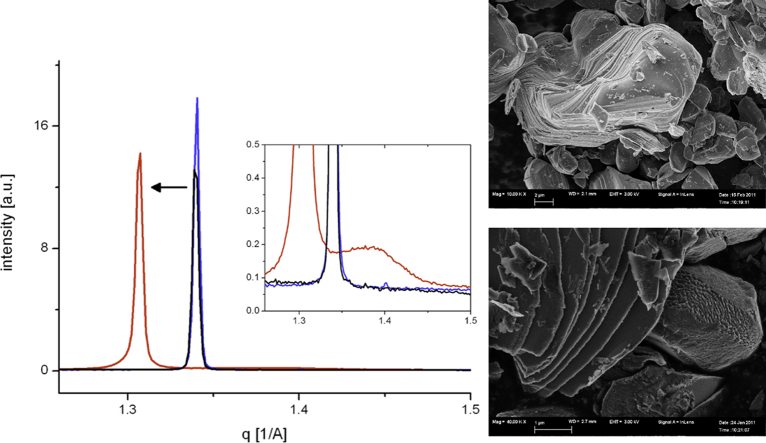

## 1 Introduction

This paper explores the changes in crystal structure and texture in powdered lithium cobalt oxide after exposure to dilute hydrochloric acid to gradually remove both lithium and cobalt. Chemical methods follow both ions in the extracting solution and the effect on lattice parameters, occupancies and temperature factors as well as the crystal texture and compared with those from electrochemical delithiation using known data [1].

In 1980 Goodenough et al. reported a new layered compound that is capable of reversibly intercalating Li-ions at 4 V: lithium cobalt oxide (${\mathrm{LiCoO}}_{2}$) [2]. Nowadays ${\mathrm{LiCoO}}_{2}$ is the most widely used cathode material for Li-ion batteries because it outrules other materials in terms of cost and performance [3]. The material has now reached energy densities in excess of 150 Wh/kg and 350 Wh/l cycle lives in excess of 1000 cycles and low self discharges <3%/month (Chapter 11 of [4]).

Both texture and chemistry are important in the behaviour of ${\mathrm{LiCoO}}_{2}$ in battery operation for powdered ${\mathrm{LiCoO}}_{2}$. The valence electronic structure formed by transition metal and oxygen ions is flexible, therefore a variation in Li concentration is possible. Delithiation (which scales the oxidation state of Co) leads to an increased covalency of the Co-bonds. (Chapter 2 of [4]). XRD measurements of chemically delithiated ${\mathrm{Li}}_{x}{\mathrm{CoO}}_{2}$ reveal at *x*=1 a hexagonal (I) at 0.92>0.76 a hexagonal (I and II) and at 0.71>0.53 a hexagonal (II) while the hexagonal (I) phase shows ionic character for lithium indicated by NMR measurements [5]. Below *x*=0.75 ${\mathrm{Li}}_{x}{\mathrm{CoO}}_{2}$ exhibits metallic properties and above this semiconductor properties [6]. A metal–insulator transition occurs in two crystallographically identical host hexagonal structures (Chapter 2 of [4]).

**Fig. 1:**
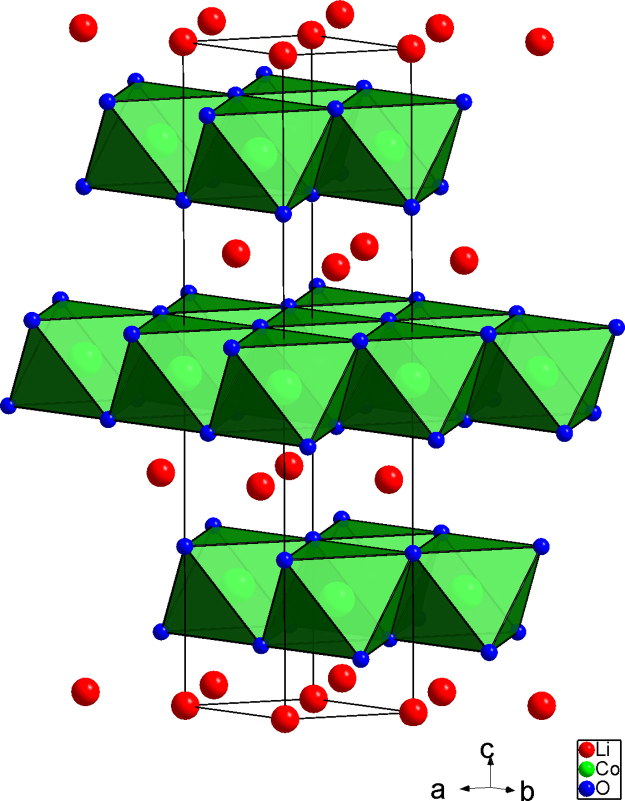
Structure of ${\mathrm{LiCoO}}_{2}$, space group *R*$\overset{¯}{3}$*m* (166).

Fig. 1 shows the crystal structure of ${\mathrm{LiCoO}}_{2}$ synthesised at high temperatures (referred to as HT-${\mathrm{LiCoO}}_{2}$) [7], [8]. The synthesis using conventional high temperature (HT) procedures results in the ideal layered *α*-NaFeO_2_-type or O3 structure (*R* $\overset{¯}{3}$*m* (166)) space group with an ABCABC stacking of layers, while low temperature (LT) synthesis results in the spinel structure (Chapter 1 of [4]) [8]. Lattice parameters for HT-${\mathrm{LiCoO}}_{2}$ are reported to be *a*=281.6 pm *c*=1405.1 pm [9] and with higher accuracy *a*=281.56(6) pm, *c*=1405.42(6) pm [1].

Electrochemically delithiated (charged) ${\mathrm{Li}}_{x}{\mathrm{CoO}}_{2}$ undergoes a phase transition from hexagonal to monoclinic and vice versa during charging and discharging when *x*=0.5 [10], [11]. In principle, delithiating is reversible up to CoO_2_ composition but the large volume changes cannot be tolerated by the particle and results in fractures and loss of contacts. During deintercalation, there is only a minor change of the lattice parameter *a* unlike *c* which exhibits expansion to up to 2–3% at *x*=0.5 [11]. The interlayer distance of the oxygen sheets (see Fig. 1) equals *c*/3. Lithium is generally completely ionised within the material and the Li-ions pull electrostatically the O–Co–O sheets together (Chapter 2 of [4]). The removal of Li^+^ causes enlargement of interlayer distances due to sheet repulsion (Chapter 11 of [4]). The lattice strain (caused by delithiation) is both anisotropic and directly correlated with the lithium concentration [11]. Another possible damage mechanism during lithiation and delithiation is differential expansion within a single grain due to Li concentration gradients generated during charging/discharging. In charging induced formation of vacancy type defects in ${\mathrm{Li}}_{x}{\mathrm{CoO}}_{2}$ indication is found such that Li^+^ reordering occurs at the limit of reversible extraction (*x*=0.55) and causes a transition from two dimensional agglomerates into one-dimensional chains [12]. At *x*_0_=1 monoclinic distortion was reported for ${\mathrm{Li}}_{x}{\mathrm{CoO}}_{2}$ with *x*=0.5 and studied by X-ray and neutron diffraction [13].

In a Li-ion battery ${\mathrm{Li}}_{x}{\mathrm{CoO}}_{2}$ can be delithiated (charged) to up to *x*=0.5 which corresponds to 140 mAh/g (theoretically 274 mAh/g) [3]. This value can be improved to 200 mAh/g (*x*=0.7) by substituting the Co in the outer layer of the core with Ti, Al or Mg (Chapter 1 of [4], [14]).

Chemical extraction of Li from ${\mathrm{Li}}_{x}{\mathrm{CoO}}_{2}$ with oxidising agents such as Cl_2_ leads to the dissolution of a part of the material, else leads to oxygen vacancies ${\mathrm{Li}}_{x}{\mathrm{CoO}}_{2-\delta }$ and to a disproportion of Co^3+^ to Co^2+^ and Co^4+^ [15]. However, chemically deintercalation by HCl characterised by X-ray absorption spectroscopy reveals that Co ions remain mainly unaffected by Li-deintercalation [16]. A weak antiferromagnetic coupling at *x*=0.65 and that the lithium oxide layer expands perpendicular to the basal plane and Li ions displace from their octahedral sites with decreasing *x* are found by neutron diffraction [17].

Extracting Li-ions chemically from O3-type ${\mathrm{LiCoO}}_{2}$ with an oxidiser leads to the formation of P3-type ${\mathrm{CoO}}_{2-\delta }$, with lower *c*-parameter than the initial crystal structure due to decreased charge on the oxide and the formation of oxygen vacancies [6]. The decrease in *c* is attributed to the decreased charge on the oxide ions and the formation of oxygen vacancies.

The shapes of CoO_6_ octahedra, studied by powder neutron diffraction are reported to be critically dependent on the distribution of sodium ions in the intervening layers [18]. However, comparison between ${\mathrm{Li}}_{x}{\mathrm{CoO}}_{2}$ and ${\mathrm{Na}}_{x}{\mathrm{CoO}}_{2}$ shows that the changes in the CoO_2_ layer are relatively rigid for the lithium compound [17]. The crystal structure of three layer ${\mathrm{Na}}_{x}{\mathrm{CoO}}_{2}$ studied by neutron diffraction for *x*=0.92 and 0.32 is trigonal; the intermediate compositions are reported to be monoclinic [19].

Periodic changes in the chemical potential (for graphite intercalation) have been successfully attributed to the propagation of strain fields along the *c*-axis of graphite [20]. The layer lattice of ${\mathrm{LiCoO}}_{2}$ as well as the crystal strains and texture changes caused by de-lithiation resemble those found in graphite intercalation [21], where the strains are strong in the *c*-axis direction and weak in the planes. Here we explore this analogy. For graphite intercalates, incommensurate layer structures and in-plane super-lattices [22] arise from changes in the metal/graphite stoichiometry and the alkali metal size. We have searched this phenomenon using neutron diffraction to emphasise the lithium scattering but only a hint of this behaviour has been found. Thus, our interest focuses on the ${\mathrm{LiCoO}}_{2}$ *c*-axis and the extent of particle size and strain broadening.

## 2 Experimental

### 2.1 Chemical de-doping

Commercially available ${\mathrm{LiCoO}}_{2}$ (Sigma Aldrich No: 442704-100G-A) here named as LCO0 was used. The chemically delithiated samples (namely LCO1, LCO2, LCO3, LCO4, LCO5, LCO6, LCO7 and LCO8) are prepared by stirring 5 g (LCO0) in 500 ml solution. The treating times, concentration of solutions, supernatant and chemical composition after delithiation are presented in Table 1. Acid solutions were prepared by diluting conc. HCl (37%) in milliQ deionised water. After this treatment, the filtered samples were washed several times with copious amounts of milliQ deionised water to remove all possibly formed LiCl and CoCl_2_ before drying at 100 °C.

**Table 1:** Chemical composition after chemical delithiation of ${\mathrm{LiCoO}}_{2}$.

Sample	Solution	Time	Li:Co in bulk	% at. Co from bulk in supernatant	% at. Li from bulk in supernatant
LCO0	–	–	0.9326	–	–
LCO1	water	1 week	0.93171	0.5138	1.1305
LCO2	1 M HCl	10 min	0.8500	4.523	6.040
LCO3	1 M HCl	30 min	0.8312	8.606	16.76
LCO4	0.1 M HCl	1 week	–	2.170	5.661
LCO5	0.75 M HCl	1 week	0.8372	5.7979	13.79
LCO6	0.5 M HCl	1 week	0.8957	10.84	24.59
LCO7	2 M HCl	17 h	0.3918	37.18	70.78
LCO8	1 M HCl	22 h	0.3425	37.41	76.56

### 2.2 Methods

The supernatant as well as the bulk was analyzed by using Inductively Coupled Plasma Atomic Emission Spectroscopy (ICP-OES). The SE micrographs were done on a ZEISS ultra plus using an extra high tension (EHT) of 3 kV an aperture of 7.5μm and a working distance of 2.4 mm.

Samples LCO0, LCO1 and LCO_8_ were investigated by three distinct diffraction techniques:

(a) Standard XRD pattern (here named Bruker data) samples were recorded on a Bruker D8Advance in a range from 16 to 130° 2*θ* using a stepwidth of 0.01537°/step and 12 s/step and Cu Kα radiation.
(b) Synchrotron Wide Angle X-ray Scattering (here named SWAXS data) samples were transferred into a 1 mm capillary and measured in transmission mode at the SWAXS beamline of the Australian Synchrotron (Melbourne), using simultaneously a SAXS and rotated WAXS detector. Wavelengths used were 1.5213 and 0.61993 Å, whereby the former wavelength was absorbed by the material, and resulted in zero scattering at all angles.
(c) Neutron diffraction data (here named Echidna data) were obtained from OPAL/ ANSTO (Sydney, Australia). The samples were loaded into 6 mm diameter cylindrical vanadium containers and measured using the Echidna configuration [23] at a wavelength of 1.6213(2)  Å.

The XRD (Bruker) data and neutron (Echidna) data were analyzed by Rietveld profile analysis [24] using the GSAS suite of programs [25]. The second set of X-ray data (SWAXS) has the advantage of a better signal/noise ratio than the XRD-data, which facilitates the recognition of small additional features. Furthermore, these data make it easier to detect small peak splits due to the use of a single wavelength, as compared to conventional XRD measurements which use the Cu *K*_*α*1_ and Cu *K*_*α*2_ lines. On the other hand, the instrument setup used is not designed to be a diffractometer, and therefore the obtained lattice parameters are less accurate than from XRD measurements.

## 3 Results

### 3.1 Composition and morphology after chemical de-doping

**Fig. 2:**
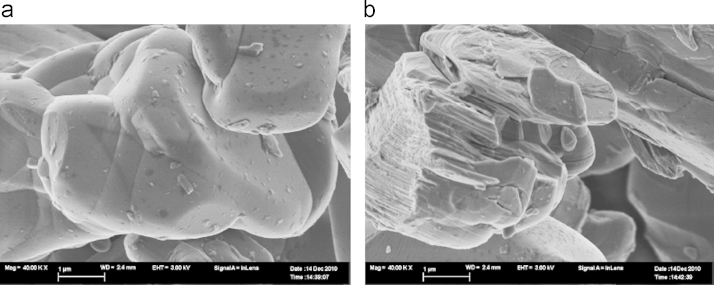
Morphology of LCO0 ${\mathrm{LiCoO}}_{2}$, the scale bar is 1μm.

The untreated ${\mathrm{LiCoO}}_{2}$ (LCO0) appears to have mostly a smooth (probably amorphous or very fine grained) surface as depicted in Fig. 2a. However, in some areas texturing of the crystal edges is found: a layered, more crystalline structure as shown in Fig. 2b. The distance between the striations (light coloured bands) is about 930 nm. Divided by the Li–Li distance of 468 pm this corresponds to about 2000 layers.

**Fig. 3:**
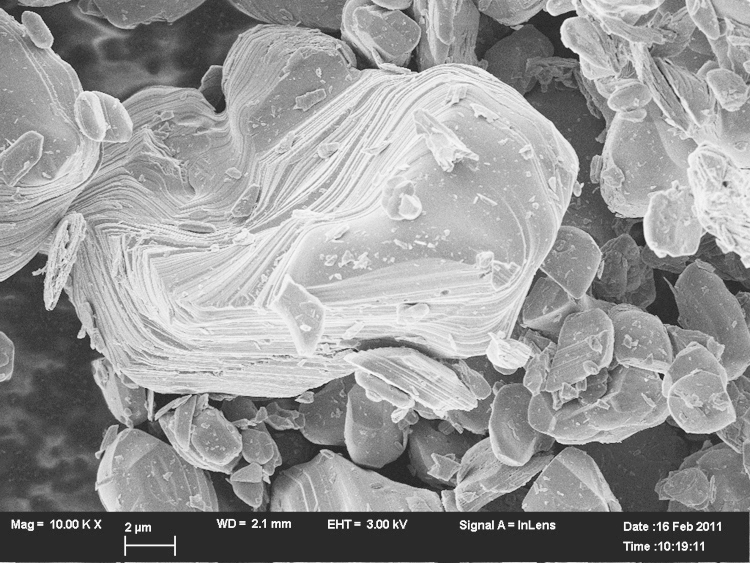
Morphology of ${\mathrm{LiCoO}}_{2}$ LCO1, the scale bar is 2μm.

Water washing of the crystals for one week at 25°C gave the pristine crystals shown in the electron micrograph (Fig. 3).

The material was delithiated by treating it with distilled water or diluted HCl (see Table 1 for treating conditions etching times, concentration and sample names). It was observed that less than a minute in conc. HCl dissolved the material to give a pink solution, while 2 ml water and 4 drops of conc. HCl (about 1 mol/l) dissolved the material to give a blue solution.

Our chemical analysis reveals preferred leaking on Li. Water alone dissolves about 0.5% Co of the material and about twice as much Li which results in a chemical composition of ${\mathrm{Li}}_{0.93}{\mathrm{CoO}}_{2}$. The analysis of the investigated samples in Table 1 shows Li depletion in the supernatant after chemical delithiation of about [Li]=2[Co].

**Fig. 4:**
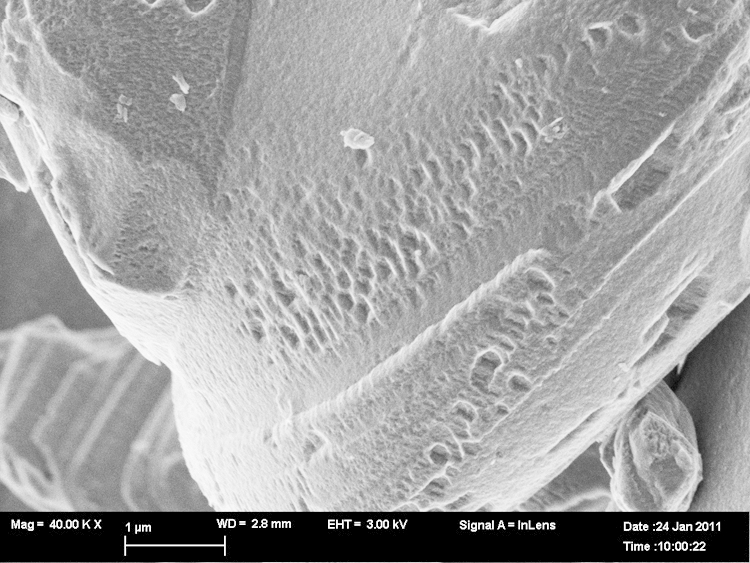
Morphology of ${\mathrm{LiCoO}}_{2}$ LCO3 the scale bar is 1μm.

**Fig. 5:**
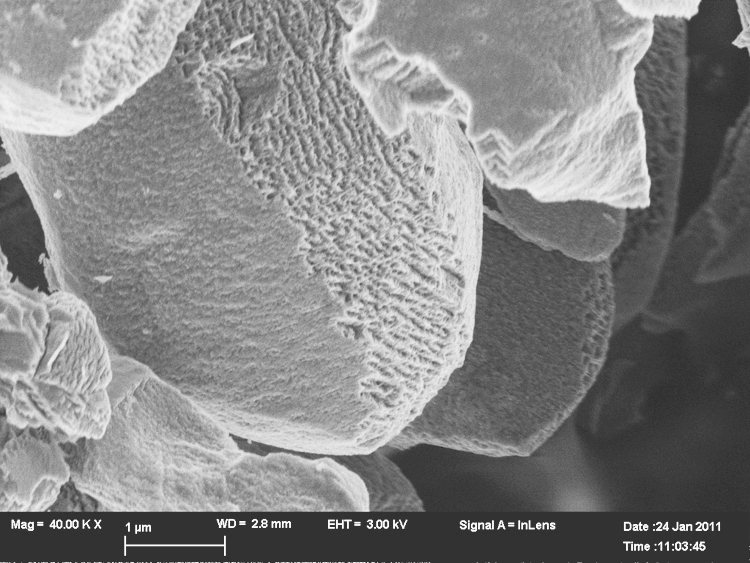
Morphology of ${\mathrm{LiCoO}}_{2}$ LCO7 the scale bar is 1μm.

The distance of the striations seen in Figs. 2b or 3 is about the same distance as the pitting pattern that is found in LCO3 depicted in Fig. 4 that emerges under acid conditions (1 mol/l). The pitting pattern found in LCO3 (corresponding to ${\mathrm{Li}}_{0.83}{\mathrm{CoO}}_{2}$) and the honeycomb-like pitting structure (Figs. 4 and 5) suggest that there may be some ordering of places on the crystal surface for the preferred leaching.

**Fig. 6:**
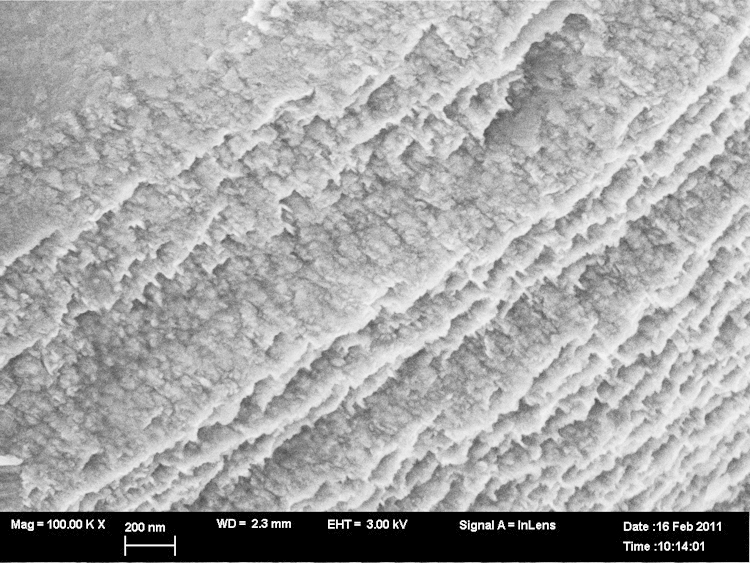
Morphology of ${\mathrm{LiCoO}}_{2}$ LCO8, the scale bar is 200 nm.

**Fig. 7:**
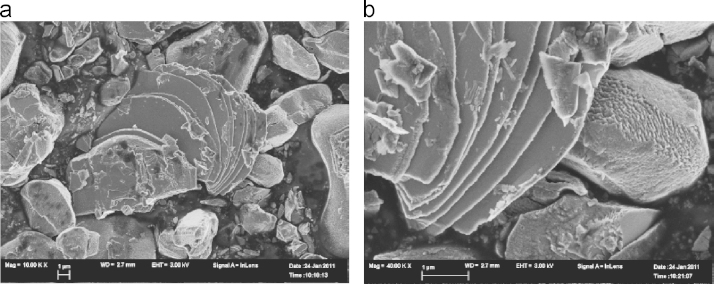
${\mathrm{LiCoO}}_{2}$ exfoliates when chemically de-doped to ${\mathrm{Li}}_{0.34}{\mathrm{CoO}}_{2}$ LCO8, the scale bar is 1μm.

The changes in morphology due to the preferred Li-pitting are extensive under higher, longer acid conditions (see Figs. 5 and 6) and finally lead to an unexpected exfoliated texture of ${\mathrm{LiCoO}}_{2}$ for LCO8 (Fig. 7a and b). This is associated with chemical de-doping to ${\mathrm{Li}}_{0.34}{\mathrm{CoO}}_{2}$ (to a stoichiometry of ${\mathrm{Li}}_{x}{\mathrm{CoO}}_{2}$ with *x*=1/3).

Water treatment of ${\mathrm{LiCoO}}_{2}$ dissolves ${\mathrm{Li}}^{+}$ and decreases the Li-content. This may cause the repulsion between the Co–O layers (see Fig. 1) to increase. However, the decrease in positive charge resulting from the loss of ${\mathrm{Li}}^{+}$ would need to be counterbalanced either by oxidation of Co(III) to Co(IV) or by the loss of O. The Li-ions could also be replaced via ion-exchange by $H^{+}$ resulting in $\mathrm{Co}{\left(O,\mathrm{OH}\right)}_{6}$ octahedra in the ${\mathrm{CoO}}_{2}$ layers.

### 3.2 Crystal structure

HT-${\mathrm{LiCoO}}_{2}$ (rhombohedral) and LT ${\mathrm{LiCoO}}_{2}$ (spinel) X-ray diffraction patterns look very similar. In LT-${\mathrm{LiCoO}}_{2}$ the *c*/*a* equals 4.90 whereas the ratio is closer to 5 for the rhombohedral layered structure (Chapter 3 of [4]). In powder XRD HT- and LT-${\mathrm{LiCoO}}_{2}$ can be distinguished by the high intensity of (003) peak and the clear splitting between (006)/(102) and (108)/(110) peak for the rhombohedral structure while the spinel structure has single (222) and (440) reflection, respectively [30]. Reimers and Dahn describe the changes in structural parameters (lattice constants *a* and *c*) upon de-lithiation as a function of the lithium concentration, *x*, in ${\mathrm{Li}}_{x}{\mathrm{CoO}}_{2}$ in a phase diagram [11].

**Fig. 8:**
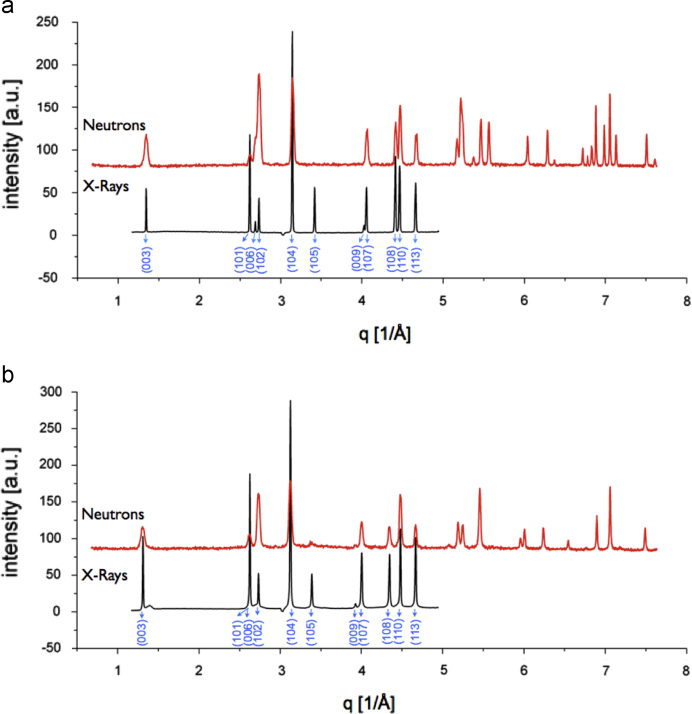
Comparison between neutron diffraction (Echidna data) and indexed synchrotron X-ray (SWAXS data). (a) LCO0 and (b) LCO8.

The X-ray diffraction patterns of LCO0 (with an occupancy of *x*=1) clearly reveal (006)/(102) and (108)/(110) peak splitting (see Fig. 8) which confirms the presence of a layered structure of HT-${\mathrm{LiCoO}}_{2}$, a lattice parameter ratio of *c*/*a*=0.499 and an excellent agreement with HT-${\mathrm{LiCoO}}_{2}$ reported in the literature from Takahashi et al., *a*=281.56(6) pm, *c*=1405.42(6) pm [1]. The Rietveld refinement of LCO0, LCO1 and LCO8 (Bruker data) is depicted in Fig. 11, Fig. 12, Fig. 13, respectively, and the obtained parameters are summarised in Table 2. The crystal structure is also consistent with data for the material “Selectipur” (Merck) described in [14], [26].

**Table 2:** Crystallographic and Rietveld structure refinement parameters of the samples LCO, LC1 and LC8 (Bruker data).

*Data collection*			

Phase Name	${\mathrm{LiCoO}}_{2}$	${\mathrm{LiCoO}}_{2}$	${\mathrm{LiCoO}}_{2}$
Sample ID	LCO0 untreated	LCO1 water treated	LCO8 1 m HCl treated
Wavelength	Cu Kα	Cu Kα	Cu Kα
Temperature (K)	293(2)	293(2)	293(2)
Specimen size (mm^2^)	12×12	12×12	12×12
Particle morphology	Platelets	Platelets	Platelets
Specimen mounted in reflection mode			
Measurement range	2*θ*min=16°	2*θ*min=16°	2*θ*min=16°
	2*θ*max=130°	2*θ*max=130°	2*θ*max=130°
Increment (deg)	0.015	0.015	0.015
hkl range	*h*=±2	*h*=±2	*h*=±2
	*k*=±2	*k*=±2	*k*=±2
	*l*=±15	*l*=±15	*l*=±15

*Refinement*			

R-Bragg (%)	0.384	0.695	0.195
R weighted profile (%)	10.57	17.03	13.43
GOF	1.26	2.31	1.29
Scale	0.0653(3)	0.1096(8)	0.0667(4)
Background	Polynome	Polynome	Polynome
	15th degree	15th degree	15th degree
Absorption correction	None	None	None
Peak type	Modified pseudo-Voigt:	a+b⁎tan(θ)+c/cos(θ)	
FWHM Gaussian			
*a*	0.000(12)	0.001(40)	0.000(37)
*b*	0.035(11)	0.045(37)	0.084(28)
*c*	0.001(16)	0.001(48)	0.001(45)
Lorentzian			
*a*	0(1)	0(2)	0(2)
*b*	2(4)	2(2)	2(6)
*c*	0(2)	0(2)	0(2)
*w(i)*=1/ $l_{\mathrm{obs}(i)}$ at each point *i*			
Number of parameters	30	30	30
Preferred orientation correction	March–Dollase		
(Dir 1 : 0 0 1)	0.869(2)	0.628(1)	0.968(3)

*Crystal data*			

Lattice parameters			
a (pm)	281.555(1)	281.539(2)	280.875(3)
c (pm)	1404.952(5)	1404.84(1)	1442.46(2)
Cell volume (pm^3^)	96.4537(9)	96.435(2)	98.551(3)
Cell parameters	31 Reflections	31 Reflections	31 Reflections
Symmetry	Rhombohedral, *R*$\overset{¯}{3}$*m*	Rhombohedral, *R*$\overset{¯}{3}$*m*	Rhombohedral, *R*$\overset{¯}{3}$*m*
Formula units	*Z*=3	*Z*=3	*Z*=3
Molecular weight (g/mole)	97.24	97.16	92.20
Crystal density (g/cm^3^)	5.022(7)	5.019(9)	4.696(11)
Crystal linear absorption coeff. (1/cm)	997.5	997.7	976.2

The lithium occupancy is a crucial parameter, but notoriously difficult to determine by powder X-ray diffraction, due to the small scattering contribution of light elements such as Li [27]. Neutron scattering is an especially attractive method to determine light elements in the presence of heavy atoms. The neutron scattering length *b* is −0.233 for ^7^Li and +0.25 for Co [we used the scattering length of −1.90 fm for Li] and provides a much higher contrast than diffraction patterns obtained by X-ray diffraction [28].

Fig. 8a and b shows a comparison between the measured neutron diffraction (Echidna) and synchrotron X-ray (SWAXS) data for the starting sample LCO0, and the partially de-lithiated sample LCO8, respectively. The data are plotted as a function of the diffraction momentum transfer q=(4πsinθ)/λ where *θ* and *λ* are the Bragg angle and the radiation wavelength, respectively. As expected, the peak positions in X-ray (SWAX) and neutron (Echidna) for each sample are identical, but the intensities differ due to the different contrast conditions.

**Fig. 9:**
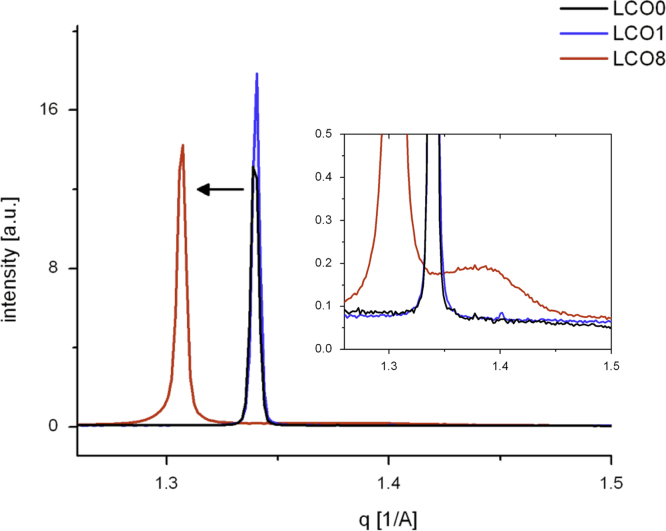
Effect of chemical dedoping leads to significant change in (003) peaks. Inset: close up of (003) peak, X-ray data (SWAXS).

Fig. 9 illustrates the significant change in the (003) peaks (SWAX data), representing an increase in the layer spacing observed after chemical de-lithiation to a chemical composition of ${\mathrm{Li}}_{0.34}{\mathrm{CoO}}_{2}$ (stochiometry of about Li 1/3) that finally leads to exfoliation of ${\mathrm{LiCoO}}_{2}$ (see Fig. 7a and b). The preferred (001) orientation in the samples was taken into account and corrected using the March–Dollase function of GSAS during the refinements. The positional, occupancy and thermal parameters of samples LCO0, LCO1 and LCO8 are summarised in Table 3.

**Table 3:** Positional, occupancy and thermal parameters of ${\mathrm{LiCoO}}_{2}$ samples LCO0, LCO1 and LCO8 (Bruker data) obtained by Rietveld refinement.

Site	Multiplicity	*x*	*y*	*z*	Occupancy	$B_{\mathrm{eq}}$
LCO0 – untreated						
Li	3	0	0	0	0.909(21)	0.48(26)
Co	3	0	0	1/2	1	0.186(25)
O	6	0	0	0.2394(1)	1	1.080(43)

LCO1 – water treated						
Li	3	0	0	0	0.898 (22)	1.502 (30)
Co	3	0	0	1/2	1	1.013 (43)
O	6	0	0	0.2403(1)	1	1.534(61)

LCO8 – 1 m HCl treated						
Li	3	0	0	0	0.283(32)	0.9(14)
Co	3	0	0	1/2	1	0.367(33)
O	6	0	0	0.2348(2)	1	1.139(60)

For the Rietveld refinement of the neutron data, we started with refining *a*, *c* and z(O), and then also the lithium occupancy (Table 4). Due to a strong correlation between the lithium occupancy and the thermal motions the latter needed to be fixed and were initially set to a value of $U_{\mathrm{iso}}=0.005\;10^{4}\;{\mathrm{pm}}^{2}$ (Table 4). However, Li is expected to be much more mobile than Co and O. Therefore, alternatively we fixed $U_{\mathrm{iso}}$ for Li, Co and O independently to known values from the literature [1], which results in more accurate values for the Li occupancy (Table 5). In fact there is an excellent agreement between our data and a very careful single crystal study [1]. It is also expected that Li is more mobile in the *xy*-plane than in the *z*-direction, but allowing for anisotropic motion did not improve the quality of the fit. Note that the fit for *a* and *c* is not dependent on other parameters. The lattice parameters *a* and *c* are in good agreement for the X-ray and neutron data. Selected interatomic distances of compounds LCO0, LCO1 and LCO8 from Rietveld refinement (Echidna and Bruker data) are presented in Table 6, Table 7.

**Table 4:** Lattice parameter (LP) and selected crystallographic data of ${\mathrm{LiCoO}}_{2}$ (Echidna data) obtained by Rietveld refinement.

Sample	LP (pm)		*z*(O)	Li occupancy	*χ*^2^
	*a*	*c*			
LCO0	281.520(3)	1404.86(3)	0.23946(5)	0.895(9)	1.732
LCO1	281.525(3)	1404.83(3)	0.23966(5)	0.883(9)	2.016
LCO8	280.822(4)	1441.96(5)	0.23432(8)	0.302(13)	2.418

**Table 5:** Lattice parameter (LP) and selected crystallographic data of ${\mathrm{LiCoO}}_{2}$ (Echidna data) obtained by Rietveld refinement temperature factors $U_{\mathrm{iso}}$ from [1].

Sample	LP (pm)		$U_{\mathrm{iso}}\;(10^{4}\;{\mathrm{pm}}^{2})$			*z*(O)	Li occupancy	*χ*^2^
	*a*	*c*	Li	Co	O			
LCO0	281.520(3)	1404.86(3)	0.012	0.00326	0.0047	0.23942(5)	0.987(10)	1.799
LCO1	281.525(3)	1404.83(3)	0.012	0.00326	0.0047	0.23962(5)	0.975(10)	2.1
LCO8	280.822(3)	1441.94(4)	0.028	0.00553	0.0065	0.23431(8)	0.366(15)	2.287

**Table 6:** Selected Interatomic distances in pm in the samples LCO0, LCO1 and LCO8 (Echidna data).

Bond	LCO0	LCO1	LCO8
Li–Li	495.7	495.7	507.3
Co–Co	495.7	495.7	507.3
O–O (over Li)	309.4	309.8	328.4
O–O (over Co)	261.3	261.6	253.7
Li–Co	285	285	289.9
Li–O	209.3	209.2	216
Co–O	192.1	192.2	189.2

**Table 7:** Selected Interatomic distances in pm in the samples LCO0, LCO1 and LCO8 (Bruker data).

Bond	LCO0	LCO1	LCO8
Li– O	209.4(1)	208.6(1)	215.7(2)
Co– O	192.0(1)	192.6(1)	189.6(1)
Li–Li (interlayer)⁎	495.73	495.69	507.43
Li–Co	285.05	285.03	289.99
O–O (Li layer)	310.0(3)	307.9(3)	327.4(3)
O–O (Co layer)	261.1(3)	263.0(3)	254.7(4)

⁎ Co–Co (interlayer distance).

**Fig. 10:**
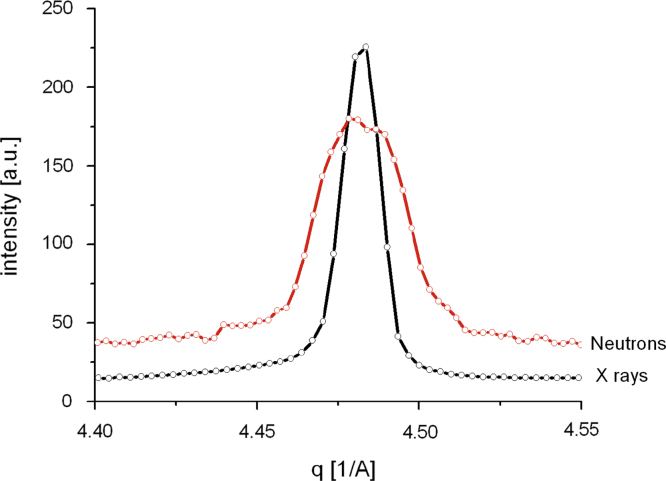
Chemical dedoping leads to small peak broadening in the neutron (Echidna) but not the X-ray (SWAXS data). Zoom on (110) reflection.

The resulting fits of the neutron data are reasonably good, but in LCO8 there seem to be significant deviations: the intensity of some peaks cannot be accounted for, and at close inspection, some or all peaks could be slightly broadened, most obviously the (102) peak and the (110) peak (as shown in Fig. 10). It is clearly visible that this peak splitting was not observed in the respective X-ray (SWAXS) data, which leads us to believe that the sample for the Echidna data set was simply more heterogeneous (much larger sample size) than the sample for the SWAX data set.

**Fig. 11:**
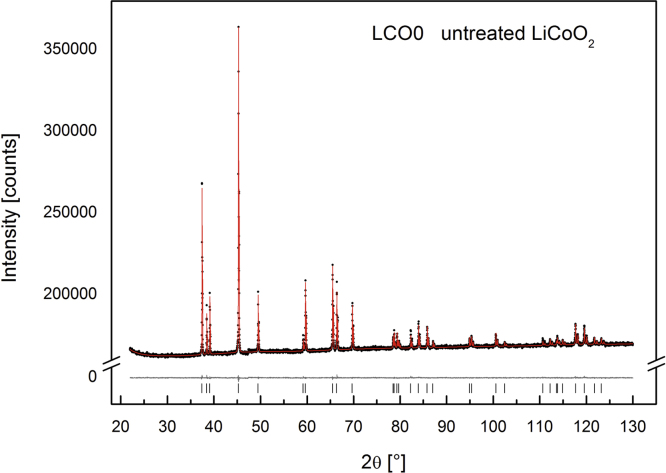
Rietveld refinement for LCO0 (Bruker data).

**Fig. 12:**
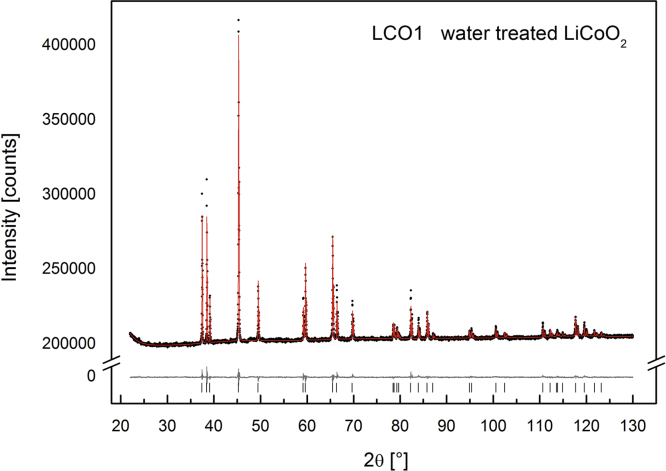
Rietveld refinement for LCO1 (Bruker data).

**Fig. 13:**
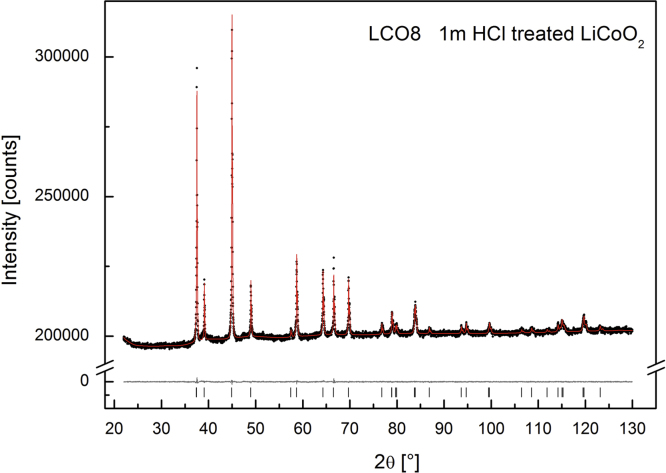
Rietveld refinement for LCO8 (Bruker data).

**Fig. 14:**
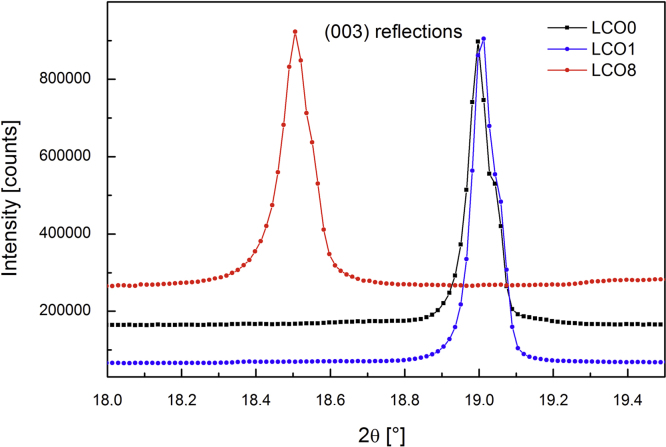
Peak shift for 00l reflections (Bruker data).

In any case, we could not attribute these deviations to a distortion of the hexagonal unit cell to monoclinic, which has been reported in ${\mathrm{Li}}_{x}{\mathrm{CoO}}_{2}$ at *x*=0.5 [11] for electrochemically de-lithiated samples. Further, we could not attribute them to a change in the Co-occupancy, to the occupation of tetrahedral sites or a partial Li-Co site exchange [31] (like 3a/3b in ${\mathrm{LiNi}}_{0.8}{\mathrm{Co}}_{0.19}{\mathrm{Cu}}_{0.01}O_{2}$ [32]), or to the formation of de-lithiated materials like ${\mathrm{Co}}_{3}O_{4}$ [33], [34] ${\mathrm{Co}}_{2}O_{3}$ [35], CoO [36] or CoOOH. However, there is an additional small broadened peak for sample LCO8 occurring at a *d*-spacing of 453 pm, that is better visible in the X-ray data (see zoom Fig. 9). This may indicate the starting formation of a new phase and will be discussed in the next section in more detail (Fig. 14).

## 4 Discussion

At the extremes of our experiment, the water washed sample LCO1 showed a slightly decreased lattice parameter *c* as compared to the starting sample LCO0 and an occupancy of ${\mathrm{Li}}_{x}{\mathrm{CoO}}_{2}$ with *x*=0.975 from a good fit in Rietveld refinement of the neutron data. The chemical analysis showed a content of *x*=0.93.

In the partially de-lithiated sample LCO8, the lattice parameter *c* significantly increased while *a* slightly decreased, in accordance to the trend generally observed for de-lithiation of ${\mathrm{LiCoO}}_{2}$ [11]. This shift in the *c*-dimension calculated from Rietveld refinement gives a change from 1405 pm (LCO0) to 1442 pm (LCO8) (for Bruker and Echidna data). At ${\mathrm{Li}}_{x}{\mathrm{CoO}}_{2}$ with *x*=0.5, a distortion from a hexagonal to monoclinic unit cell can take place [11]. This distortion would be best seen by a split (104) peak, but there is no splitting of this peak visible in our SWAXS data, showing that the hexagonal structure is retained. Rietveld refinement of this neutron data set gives a Li-occupancy of 0.37, which is in good agreement with the chemical analysis of 0.34. At this composition partial exfoliation of ${\mathrm{LiCoO}}_{2}$ sheets takes place (see Fig. 6a and b).

The SWAXS data also show that at this composition an additional small broadened peak at a *d*-spacing of 453 pm appears (see zoom in Fig. 9). From a comparison with data from literature, this is possibly the starting formation of a O1-type structure of ${\mathrm{CoO}}_{2}$, previously observed for electrochemically de-lithiated samples [38], [39], or the P3-type of ${\mathrm{CoO}}_{2}$ (lattice parameter *c* of 1330 pm) structure, previously observed for chemically de-lithiated samples [6]. The observed *d*-spacing fits better to the P3-structure, whose first peak is reported to be at a corresponding *d*-spacing of 449 pm, while that of O1 is at 422 pm. Extracting Li-ions chemically from O3-type ${\mathrm{LiCoO}}_{2}$ with an oxidiser has previously been found to lead to the formation of P3-type ${\mathrm{CoO}}_{2-\delta }$, with lower *c*-parameter than the initial crystal structure due to the decreased charge on the oxide and the formation of oxygen vacancies [6].

Generally, the agreement in the lattice parameters from neutron and X-rays is very high (Table 2, Table 4, Table 5) and the widths of the diffraction peaks (except LCO8) are nearly at the experimental resolution of the X-ray and neutron instruments ($\Delta d/d=10^{-3}$). The peak widths in the X-ray (SWAXS) data are smaller than those in the neutron (Echidna) data. Thus, conclusions about strain broadening will be made from the synchrotron data.

The peaks for LCO0, LCO1 and LCO8 have different widths. Those for LCO0, LCO1 have full widths at half maximum with $\Delta q=0.004\;{Å}^{-1}$ (1.4×10^−4^ radians). This width is approximately the resolution width of the instrument and gives a minimum crystallite coherence length (in the c direction) of ca 1.1μm using the Scherrer formula. For LCO8, the principal peak has a broad Lorentzian base and a width 20% larger than those of LCO0, LCO1 ($\Delta q=0.005\;{Å}^{-1}$ (1.7×10^−4^ radians) giving a coherence length of ca. 1.0μm. The broad component of the LCO8 diffraction at *q*=1.38 Å^−1^ (*d* spacing 4.55 Å, width 0.14 Å^−1^ (1.67×10^−2^ radians)) results in a coherence length of about 84 Å—the thickness of the two c-axis unit cells.

As the electron micrograph shows (Fig. 3), the largest crystals of ${\mathrm{LiCoO}}_{2}$ in the untreated sample are many microns big so the coherence lengths of ca. 1μm must come from the smallest crystals present, but it should be noted that the large observed grains are not necessarily single crystals. De-lithiation to a stochiometry of about 1/3 finally leads to the exfoliation of ${\mathrm{LiCoO}}_{2}$ (see Fig. 6). There are two different outcomes – a terrace of well separated sheets and etched crystals – the etching apparently occurs at the edges of planes perpendicular to the *c*-axis. Exfoliation and etching of ${\mathrm{Li}}_{0.37}{\mathrm{CoO}}_{2}$ after lithium ion extraction are shown in Fig. 7(a) and (b).

The projection shown in the electron micrograph (Fig. 7) does not allow easy determination of the thickness of the terrace layers. In the hypothesis that they are well-spaced exfoliated layers, the thickness of light bands corresponds to about 0.04μm. The average repeat on the etched pattern between light worm-like areas (assumed to be the beginning of terraces) is also about 0.04μm.

To capture the early stages of the delamination process we have taken micrographs under mild conditions of lithium extraction. There is, initially, a distinct patterning of the crystals along the planes perpendicular to the *c*-axis shown in Fig. 4. The size of the square pits here is about 0.14μm. At a later stage the higher resolution scan of a similar area of etched crystal is shown in Fig. 6. The thickness of the light bands in this projection corresponds to ca. 0.02μm and the average separation between bands is 0.07μm.

Lithium cobalt oxide crystals lose lithium under mild acid conditions in a progressive way until a stoichiometry of about ${\mathrm{Li}}_{x}{\mathrm{CoO}}_{2}$ with *x*=0.34 causes exfoliation. In this process structures in the etched interface of 20–80 nm develop periodic chemical weakness in about this sort of interval along the *c*-axis. Such periodic changes in the chemical potential (for graphite intercalation) have been successfully attributed to the propagation of strain fields along the *c*-axis of graphite [20].

The idea is that the chemical attack at the surface of the intercalation compound ${\mathrm{Li}}_{x}{\mathrm{CoO}}_{2}$ (the start of delithiation in a particular layer) produces a local strain field, which because of the elasticity of the crystal may propagate tens of hundreds of angstrom from the “attack” site making in-between layers less liable to reaction because the layers are slightly closed. The layer lattice and its elasticity in ${\mathrm{Li}}_{x}{\mathrm{CoO}}_{2}$ along the *c*-axis makes this suggestion plausible.

Safran and Hamman׳s paper [20] describes quantitatively the interaction energies of islands of intercalate through the coherent strains that they introduce into the layer lattice. The interaction is logarithmically dependent on the separation of the islands so long as the islands are big enough compared to the distance apart. The theory not only provides a good description of staging and mixed intercalate formation but also demonstrates that for a fixed concentration of intercalate per layer a pure-stage configuration is the most stable.

Such an explanation could apply to both the formation of sheet like exfoliated structures (Fig. 7) (especially if the last events of lithium release are fast) and the banding as etching progresses.

## 5 Conclusion

The delithiation of lithium cobalt oxide crystals in aqueous environments studied by high resolution X-ray and neutron diffraction with Rietveld refinement has shown some of the changes in crystal structure and texture that result. There was robust conservation of the structure of ${\mathrm{Li}}_{x}{\mathrm{CoO}}_{2}$ until just above a lithium stoichiometry of ca. *x*=0.34 when exfoliation occurred. The exfoliated lamellae were imageable in the scanning electron microscope with a thickness of 0.04 μm. Similar thicknesses were seen in the etching process and we have related these phenomena to long range strain fields produced in the layer lattice by the delithiation process. These fields provide a change in local chemical potential which may be the origin of selective reactivity to delithiation. The analogy is made with graphite intercalates where such strain fields are responsible for the intercalate staging process.
